# Provider attitudes towards quality improvement for myocardial infarction care in northern Tanzania

**DOI:** 10.1371/journal.pgph.0003051

**Published:** 2024-04-04

**Authors:** Lauren A. Coaxum, Francis M. Sakita, Jerome J. Mlangi, Godfrey L. Kweka, Tumsifu G. Tarimo, Gloria A. Temu, Kajiru G. Kilonzo, David Arthur, Janet P. Bettger, Nathan M. Thielman, Alexander T. Limkakeng, Julian T. Hertz

**Affiliations:** 1 Department of Emergency Medicine, Duke University Medical Center, Durham, North Carolina, United States of America; 2 Department of Emergency Medicine, Kilimanjaro Christian Medical Centre, Moshi, Tanzania; 3 Department of Internal Medicine, Kilimanjaro Christian Medical Centre, Moshi, Tanzania; 4 Duke Global Health Institute, Duke University Medical Center, Durham, North Carolina, United States of America; 5 Department of Health and Rehabilitation Sciences, Temple University, Temple University, Philadelphia, Pennsylvania, United States of America; Tribhuvan University Institute of Medicine, NEPAL

## Abstract

**Introduction:**

Myocardial Infarction (MI) is a leading cause of death worldwide. In high income countries, quality improvement strategies have played an important role in increasing uptake of evidence-based MI care and improving MI outcomes. The incidence of MI in sub-Saharan Africa is rising, but uptake of evidence-based care in northern Tanzania is low. There are currently no published quality improvement interventions from the region. The objective of this study was to determine provider attitudes towards a planned quality improvement intervention for MI care in northern Tanzania.

**Methods:**

This study was conducted at a zonal referral hospital in northern Tanzania. A 41-question survey, informed by the Theoretical Framework for Acceptability, was developed by an interdisciplinary team from Tanzania and the United States. The survey, which explored provider attitudes towards MI care improvement, was administered to key provider stakeholders (physicians, nurses, and hospital administrators) using convenience sampling.

**Results:**

A total of 140 providers were enrolled, including 82 (58.6%) nurses, 56 (40.0%) physicians, and 2 (1.4%) hospital administrators. Most participants worked in the Emergency Department or inpatient medical ward. Providers were interested in participating in a quality improvement project to improve MI care at their facility, with 139 (99.3%) strongly agreeing or agreeing with this statement. All participants agreed or strongly agreed that improvements were needed to MI care pathways at their facility. Though their facility has an MI care protocol, only 88 (62.9%) providers were aware of it. When asked which intervention would be the single-most effective strategy to improve MI care, the two most common responses were provider training (n = 66, 47.1%) and patient education (n = 41, 29.3%).

**Conclusion:**

Providers in northern Tanzania reported strongly positive attitudes towards quality improvement interventions for MI care. Locally-tailored interventions to improve MI should include provider training and patient education strategies.

## Introduction

Myocardial Infarction (MI) is a leading cause of death and disability worldwide [1]. Over the past several decades, the development of evidence-based MI therapies and interventions, such as early administration of aspirin, percutaneous coronary intervention, and long-term dual antiplatelet therapy, have resulted in decreased mortality and morbidity, particularly in higher-income settings [1, 2]. As evidence-based care for acute MI has evolved, a large number of quality improvement studies have been conducted in high-income countries to improve uptake of evidence-based MI Care [3–6].

Although MI has historically been less of a priority for researchers and policy-makers in sub-Saharan Africa (SSA), existing evidence suggests the burden of MI in the region is large and growing [7, 8]. Few studies have been conducted to understand MI care processes in SSA. Our work in Tanzania identified several gaps in evidence-based acute MI care: electrocardiogram (ECG) testing was carried out in only half of patients presenting to the ED with acute chest pain, approximately 90% of acute MI cases were misdiagnosed, only a small portion of patients with acute MI received aspirin in the emergency department, and 43% of patients with MI died within 30 days of hospital presentation [9, 10]. Furthermore, fewer than one in ten of surviving patients with acute MI were taking evidence-based therapies such as antiplatelet agents following hospital discharge [10]. Given these findings, quality improvement interventions to increase uptake of evidence-based care are needed in Tanzania. However, two recent systematic reviews of quality improvement strategies for cardiovascular disease did not identify any such published studies carried out in low-income countries [11, 12] Although a number of interventions have been shown to improve quality of MI care in high-income settings, these strategies may not be suitable for resource-limited settings like Tanzania where substantially different facilitators and barriers to care exist [13]. There is a need to further understand these factors to provide patients with better care.

Effective, locally-tailored interventions are urgently needed to improve MI care in SSA. One of the most important components to designing an effective quality improvement program to improve the uptake of evidence-based care is understanding the attitudes of the intended target population towards such interventions. Since acute MI care takes place in hospitals and is usually driven by providers, understanding attitudes of hospital-based providers towards MI care is essential to designing an effective quality improvement intervention. To better inform the design of a planned quality improvement intervention for MI care at our facility, we conducted a survey of providers at a large referral hospital in northern Tanzania. The purpose of this study was to determine whether providers considered MI a priority and whether they would support a quality improvement intervention for MI.

## Methods

### Setting

This study was conducted at Kilimanjaro Christian Medical Centre (KCMC), a zonal referral hospital serving a population of approximately 15 million persons in the Northern Zone Tanzania. KCMC has a 24-hour Emergency Department (ED) and has capacity for ECG testing, echocardiography, cardiac biomarker assays, and x-ray imaging, but does not currently have capacity for percutaneous coronary intervention or cardiac catheterization. KCMC is staffed by a mix of general physicians with no specialty training, physicians with residency training in Emergency Medicine, and physicians with residency training in Internal Medicine. Some of the physicians are also those undergoing residency training in the Internal Medicine Department. At the time of this study, KCMC did not have any cardiologists on staff. KCMC has an MI care protocol for providers in the ED and internal medicine wards to follow. KCMC is well-stocked with basic cardiovascular medications including aspirin, clopidogrel, heparin, statins, beta-blockers, nitrates, and thrombolytics. KCMC was the site of our preliminary work which identified multiple gaps in evidence-based acute MI care [10, 14–16]. In general, patients presenting with acute MI at KCMC are first cared for in the ED and then admitted to the medical ward for inpatient care. Follow-up care for patients discharged from the hospital with MI is provided at a dedicated outpatient cardiac clinic, staffed by internal medicine physicians. The local language is Swahili, and nearly all providers at KCMC are fluent in both English and Swahili.

### Study population

Any physician, hospital administrator, or nurse working in the KCMC ED, the medical ward, or the cardiac clinic was eligible for inclusion in this study. Of note, the cardiac clinic is staffed by the Internal Medicine Department. These three areas were staffed by a total of 187 providers during the study period, including 91 nurses, 90 physicians, and 6 administrators. Administrators included charge nurses, department heads, and hospital directors, all of whom had a background in clinical care. For this reason, the term “providers” is used to describe survey respondents. Providers working elsewhere in the hospital where acute MI patients would not typically be cared for were excluded from this study. There were no other exclusion criteria.

**Study procedures.** This study was conducted from May 30^th^, 2022, through September 8^th^, 2022. Trained research assistants approached eligible staff to offer study enrollment. Research assistants generally approached staff during breaks to minimize any disruption to ongoing clinical care. Convenience sampling was used; research staff approached any eligible provider who was working in the ED, medical ward, or cardiac clinic during enrollment days. After providing informed consent, enrolled participants completed a tablet-based survey regarding attitudes towards MI care (described below). To minimize social desirability bias, participants self-administered the survey; a research assistant was present to assist in case of any issues, but the respondents’ answers were generally not visible to the research assistant. Survey completion took approximately 15 minutes; participants were reimbursed 5,000 Tanzanian shillings (approximately 2 USD) for their time.

### Survey development

The survey was developed by an interdisciplinary team of implementation scientists, emergency physicians, internal medicine physicians, cardiologists, clinical officers, and social scientists, from Tanzania and the United States. The survey was designed using the Theoretical Framework for Acceptability (TFA); [17] this framework describes acceptability of interventions as a multicomponent construct consisting of 7 core components: affective attitude, burden, perceived effectiveness, ethicality, intervention coherence, opportunity costs, and self-efficacy. Because this study was conducted prior to the development of a formal quality improvement intervention, our questionnaire only consisted of questions related to four constructs (affective attitude, burden, perceived effectiveness, and ethicality). The remaining constructs will be assessed later in our quality improvement process, after intervention design and piloting. The final survey consisted of 41 items, including a mix of multiple-choice and free response questions. Multiple choice questions included Likert scale questions eliciting opinions about MI care as well as picklists consisting of quality improvement strategies from the Expert Recommendations for Implementing Change (ERIC) project [18]. All survey questions were developed in English and then translated into Swahili and back-translated into English by members of the study team fluent in both languages. All survey questions were presented in both English and Swahili to maximize participant comprehension. The survey instrument was also pre-tested with 5 providers at KCMC to ensure comprehensibility. The full survey is provided as a supplement (see S1 Text). The survey also collected information about participant demographics, job title, level of training, and work location.

### Statistical methods

All statistical analyses were performed in the R Suite. Categorical variables are presented as frequencies and continuous variables are presented as medians (interquartile ranges). The primary outcome was the proportion of participants agreeing or strongly agreeing with the statement, “I am interested in participating in a quality improvement project to improve MI care at my facility.” Based on similar prior research regarding provider attitudes towards alcohol use disorder at KCMC, [19] we expected that 90% of participants would agree or strongly agree with this statement; in order to estimate this proportion with a 5% margin of error and 95% confidence, a sample size of at least 139 participants was needed. We also conducted a supplemental analysis to determine if there were a significant differences in the proportion of nurses compared to physicians/administrators agreeing with each individual question using Pearson’s chi-squared distribution.

### Ethics

This study received ethical approval from the institutional review boards at the Tanzania National Institute for Medical Research, KCMC, and Duke Health. All participants provided written informed consent prior to participation.

## Results

A total of 140 providers were enrolled, with a median age of 28 years (Table 1). Of participants, 73 (52.1%) were female. Most participants were nurses (n = 82, 58.6%) or general physicians (n = 51, 36.4%), and most participants worked in the Emergency Department (n = 71, 50.7%) or the inpatient medical ward (n = 61, 43.6%).

**Table 1:** Participant demographic characteristics (N = 140).

Characteristic	n	%
Female sex	73	52.1
Age, median (IQR), years	28 (23–32)	
**Provider Type:**		
General physician	51	36.4
Specialist physician (emergency medicine and internal medicine)	5	3.6
Nurse	82	58.6
Administrator	2	1.4
**Work Locations:**		
Emergency Department	71	50.7
Inpatient medical ward	61	43.6
Cardiac Clinic	8	5.7

**Fig 1:**
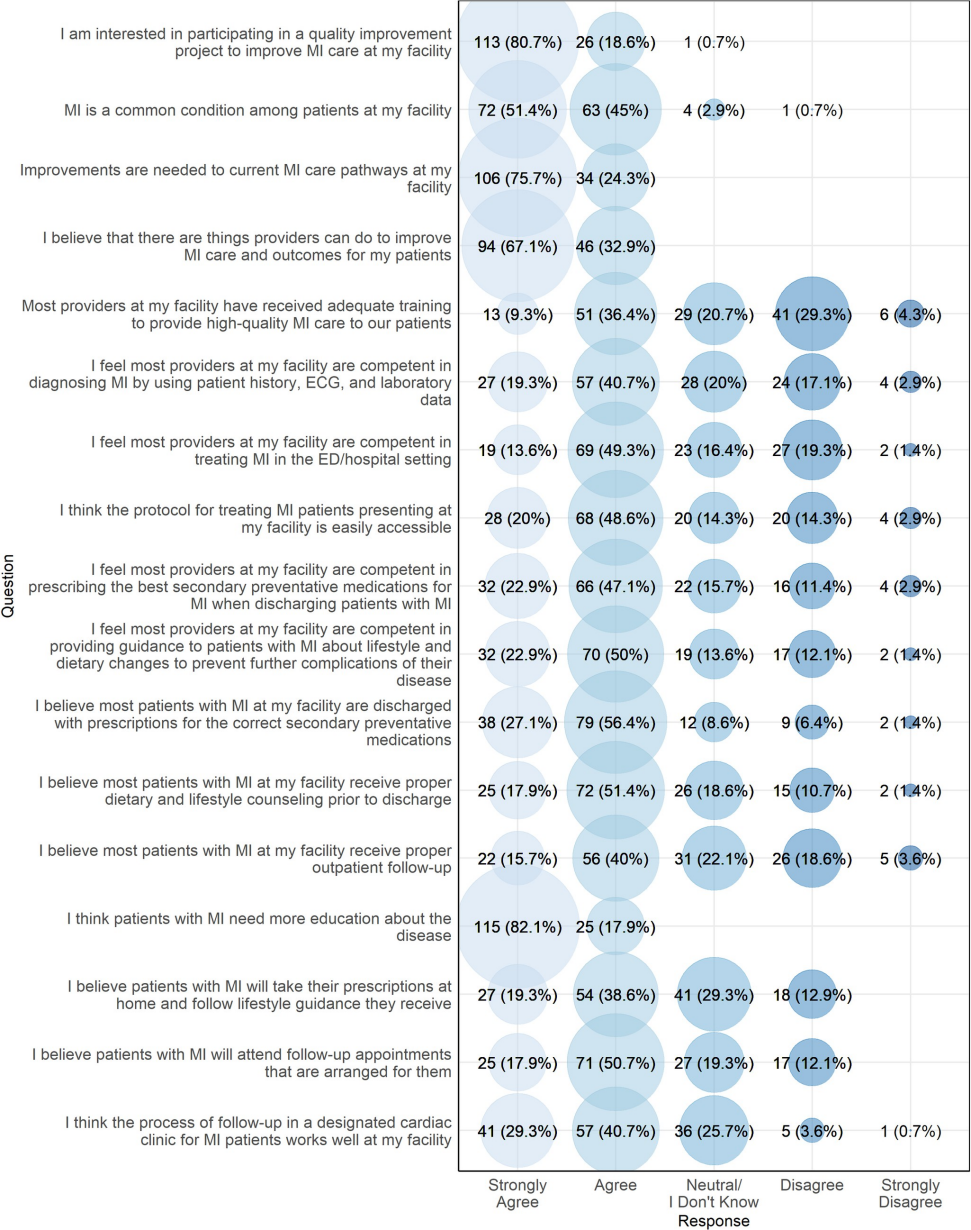
Provider affective attitudes towards MI care, northern Tanzania, 2022 (N = 140).

Fig 1 presents the affective attitudes of participants regarding MI care. Providers were interested in participating in a quality improvement project to improve MI care at their facility, with 113 (80.7%) strongly agreeing and 26 (18.6%) agreeing with this statement. All participants agreed or strongly agreed that improvements to MI care pathways at their facility were needed, and all providers either strongly agreed or agreed that patients with MI need more education about the disease. There was less consensus about whether providers had adequate training in MI, whether patients currently received appropriate inpatient and follow-up care for MI, and whether patients would adhere to outpatient medications and appointments.

**Fig 2:**
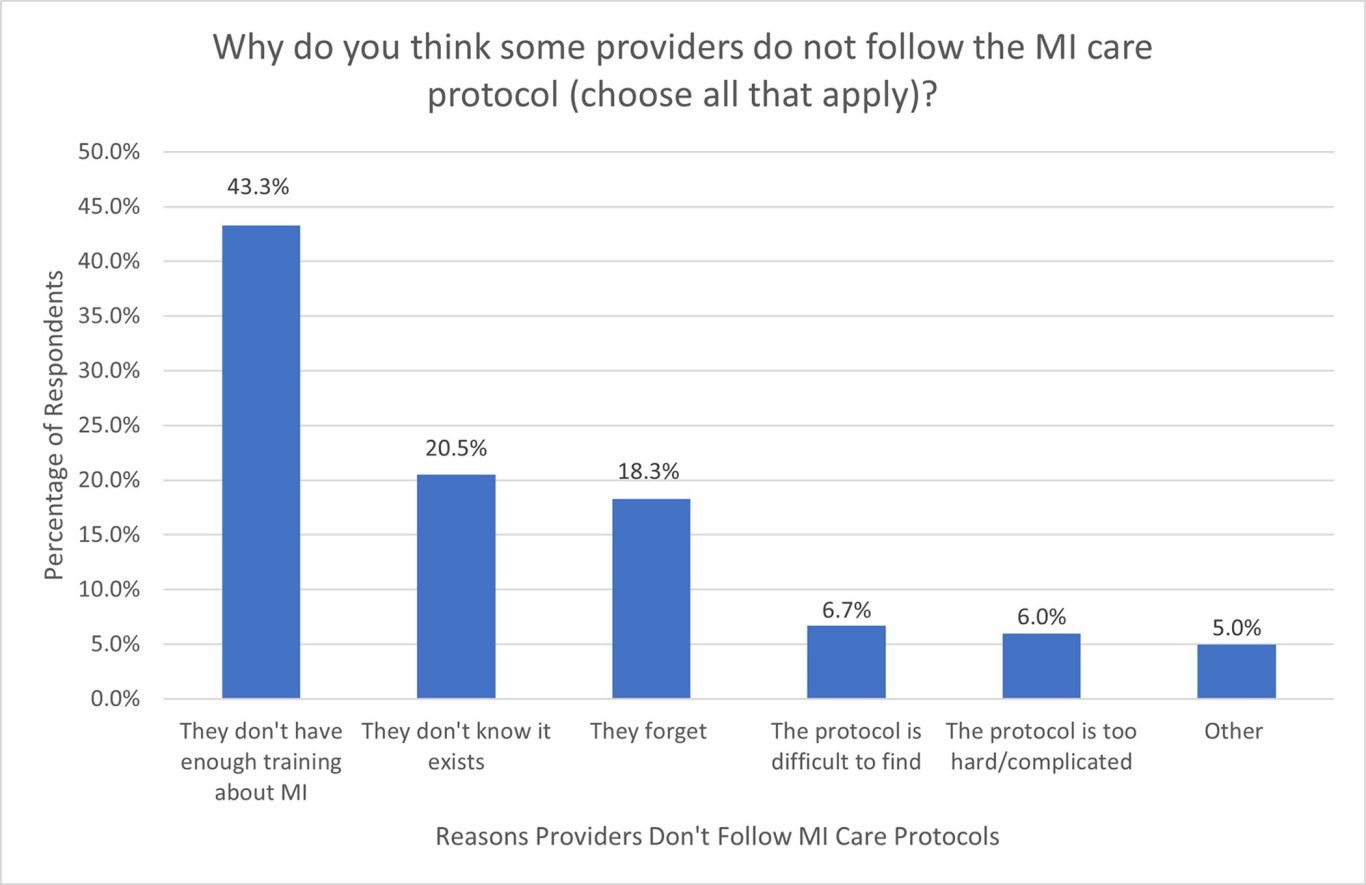
Provider responses to the question, “Why do you think some providers do not follow the MI care protocol at your facility?” participants were allowed to choose multiple answers.

Though their facility has an MI care protocol, only 88 (62.9%) providers were aware of it, with 32 (22.9%) believing that it did not exist and 20 (14.3%) being unsure whether one existed. When respondents were asked why providers did not follow the existing MI protocol, the most cited reasons were inadequate training among providers (n = 78, 43.3%), unawareness of the protocol’s existence (n = 37, 20.5%), and forgetting (n = 33, 18.3%) (Fig 2).

**Fig 3:**
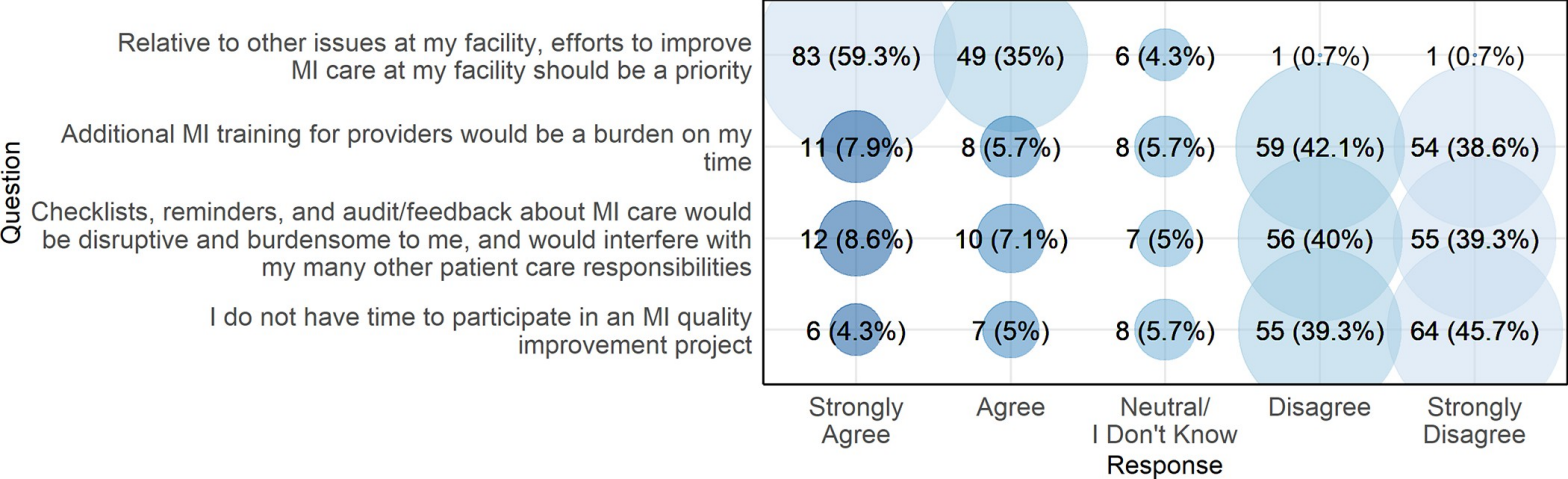
Provider perceptions of the burden associated with a quality improvement program for MI care, northern Tanzania, 2022 (N = 140).

Fig 3 presents participant responses to questions regarding the perceived burdens of a quality improvement program for MI care. Most providers (n = 132, 94.3%) either strongly agreed or agreed that, relative to other issues at their facility, efforts to improve MI care should be a priority. The vast majority of providers either disagreed or strongly disagreed that MI quality improvement work would be burdensome or disruptive to providers or patients. Overall, there were no significant differences in the proportions of nurses versus doctors/administrators agreeing with each survey item (see S1 Table).

**Fig 4:**
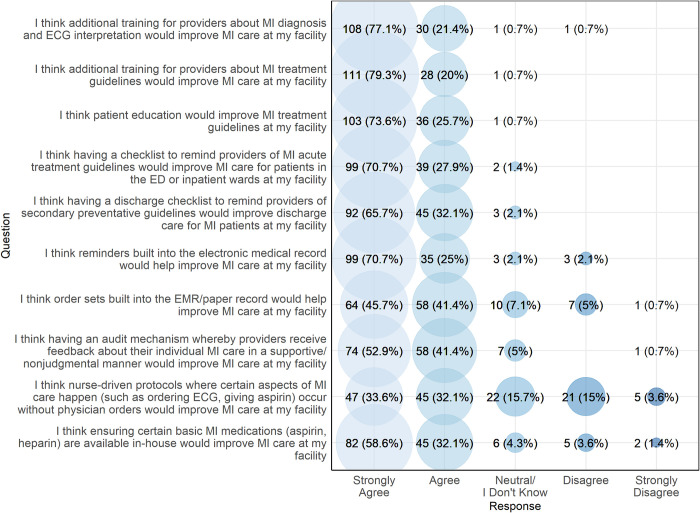
Perceived effectiveness of various MI quality improvement strategies among providers, northern Tanzania, 2022 (N = 140).

**Fig 5:**
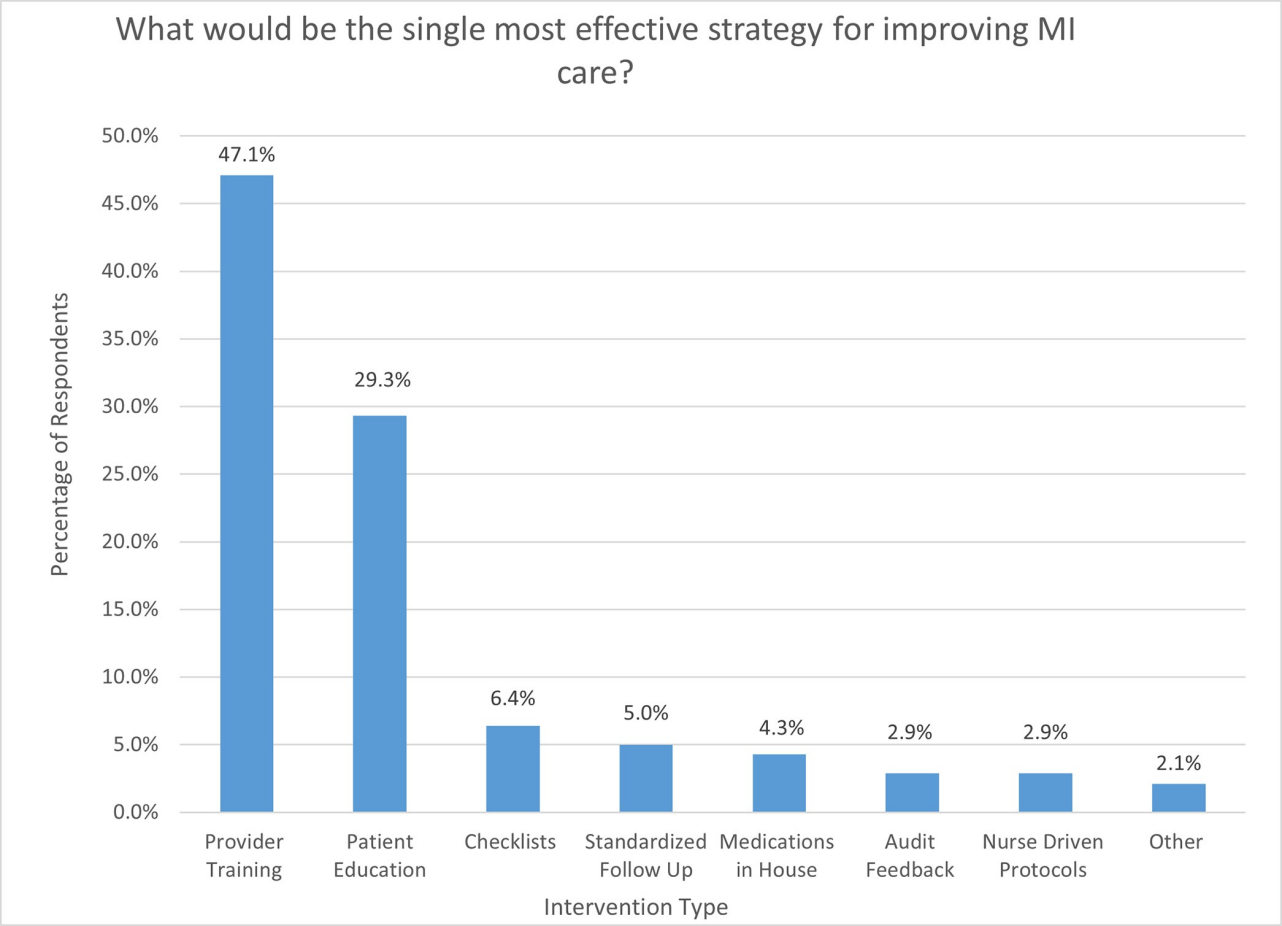
Provider responses to the question, “What would be the single most effective strategy for improving MI care at your facility?”, northern Tanzania, 2022 (N = 140).

Fig 4 presents the perceived effectiveness of various MI quality improvement strategies among providers. The majority of participants either agreed or strongly agreed that each of the listed quality improvement strategies would be effective, including provider training, patient education, checklists, reminders within the electronic medical records system, order sets, ensuring adequate supplies of medications, and audit/feedback. Respondents were asked if they preferred an audit/feedback mechanism in which feedback is given to individuals, the department, or both; 75 (53.6%) said they preferred departmental-level feedback, 26 (18.6%) preferred individual feedback, and 39 (27.9%) said they would like both types of feedback. Fewer providers (n = 92, 65.7%) thought that nurse-driven protocols would be effective. When asked to identify the single intervention that would have the greatest impact on improving MI care, participants most commonly identified provider training (n = 66, 47.1%) and patient education (n = 41, 29.3%) (Fig 5).

**Fig 6:**
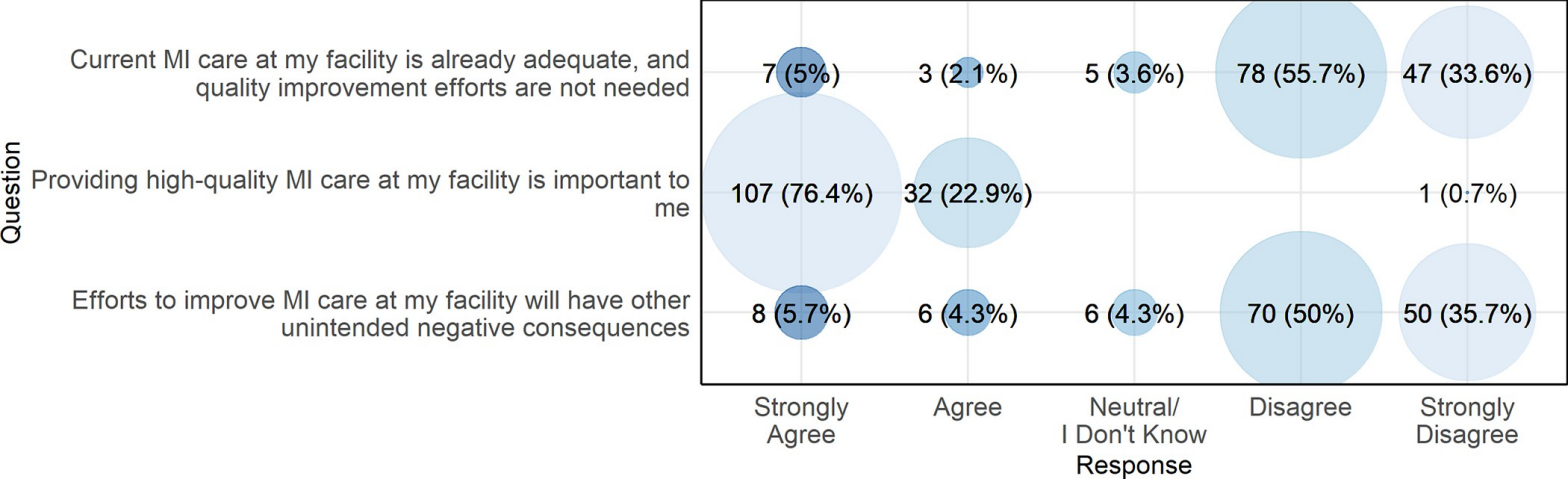
Provider perceptions of the ethicality of an MI quality improvement intervention in northern Tanzania, 2022 (N = 140).

Fig 6 presents provider perceptions of the ethicality of a quality improvement intervention for MI care in northern Tanzania. Most participants disagreed or strongly disagreed that current MI care at their facility is already adequate or that quality improvement efforts for MI care would have unintended negative consequences. The vast majority (n = 139, 99.3%) stated that providing high-quality MI care was important to them.

## Discussion

Our objective was to assess provider attitudes towards MI care quality improvement in northern Tanzania. We surveyed providers to assess four different acceptability domains, including affective attitude, burden, perceived effectiveness, and ethicality. We found strong support for MI quality improvement, with nearly all respondents expressing interest in participating in an MI care improvement intervention. We did not identify any substantial concerns regarding burden or ethicality among participants, and participants perceived a wide range of quality improvement strategies to be potentially effective in improving MI care in Tanzania. Overall, these findings indicate organizational readiness for quality improvement work from a provider perspective. These results will inform the development of a planned quality improvement intervention for MI care in northern Tanzania.

Prior research has indicated great need for improved MI care in Tanzania [9, 10]. Quality improvement for MI care has been proven to allow for better care of patients diagnosed with MI in high income countries [3–6]. Similar studies have not been conducted in low-income countries, and this study is an important first step to creating an intervention in this setting. With regards to affective attitudes, all providers agreed that improvements were needed in their MI care pathways at their facility and believed that that providers can do more to help improve MI care and outcomes for their patients.

Notably, we found that many providers were not aware of the MI care protocol at their facility, indicating an important need for ongoing training and adequate dissemination of treatment protocols. Further qualitative study is needed to explore why providers are not aware of the protocol or forget about its existence. A need for more provider training in MI diagnosis and treatment was identified by respondents in multiple sections of our survey. Respondents also felt that provider training would be the single most effective strategy for improving MI care at their facility.

Most providers thought that having checklists to guide the treatment and discharge of patients with acute MI would improve care. Nurse-driven protocols were less favorable among respondents, which may be related to different scopes of practice in Tanzania. The results of this study suggest that, at a minimum, both provider training and patient education should be included in our planned quality improvement intervention.

Importantly, not only were most providers interested in improving MI care at their facility, they also did not feel that a quality improvement intervention would be too burdensome to their workflow or disruptive to their ability to provide patient care. There is scant comparative data regarding provider attitudes towards MI care improvement elsewhere in SSA or in other low-income countries. Even in high income countries where quality improvement has been studied more extensively, provider attitudes have not been the focus.

Our study has several strengths. First, our survey was based on the Theoretical Framework for Acceptability, allowing us to explore multiple domains of acceptability [17]. This framework was developed by Sekhon et al. and was created to be applied in various settings and applied to both the recipients and deliverers [17, 20]. The framework has been applied in a variety of settings, including recent studies in SSA [21, 22]. Participants included several types of providers working in a variety of areas in the hospital, which allowed for inclusion of diverse perspectives. Our study also had some limitations. First, we recruited participants from a single tertiary care center, which limits generalizability to other settings. Providers at centers with fewer resources may have different attitudes towards MI care. Additionally, participants may have been affected by social desirability biases that may have biased them towards certain positive responses. However, we attempted to mitigate this bias by having the participants self-administer the survey so that their individual responses were anonymous. Although the financial compensation participants received was minimal, this may have also influenced their responses. We attempted to mitigate this potential source of bias by providing a very small compensation only after the survey had been completed. Finally, the psychometric properties of the survey used in this study are unknown; additional study is needed to determine the validity and reliability of this instrument.

In conclusion, providers in northern Tanzania are strongly supportive of quality improvement interventions for MI care and felt that more provider training and patient education would be particularly effective in improving care. These survey results will help inform the ongoing development of a tailored quality improvement intervention to improve uptake of evidence-based care in northern Tanzania. The particular needs identified by respondents (such as better provider training, patient education, checklists and audit/feedback) will be incorporated into the design of the intervention. Further study will be needed to determine acceptability, feasibility, and efficacy of the intervention once it is fully developed and implemented.

## Supporting information

S1 Checklist Inclusivity in global research. (DOCX)

S1 Text Provider acceptability survey. (DOCX)

S1 Data Data spreadsheet. (XLSX)

S1 Table Data tables. (DOCX)
