# Supplementary material for: Provider attitudes towards quality improvement for myocardial infarction care in northern Tanzania
Source: PLOS Glob Public Health. 2024 Apr 4;4(4):e0003051. doi: 10.1371/journal.pgph.0003051 (PMC10994299; doi:10.1371/journal.pgph.0003051)
Supplement: S1 Text — (DOCX) [file pgph.0003051.s002.docx]

MI Quality Improvement Study

Provider Acceptability Survey

Today’s date/**Tarehe ya leo:** ______

Surveyor initials/**Ufupisho wa majina ya mchunguzi:**____________

Respondent provider type/**Daraja/Elimu ya mtoa huduma anayejibu:**

□ Specialist MD, specify/ **Dactari bingwa, Taja/ainisha**: ____

□ General Practitioner MD/**Daktari wa kawaida**

□ CO

□ RN

□ Administrator/ **Kiongozi**

Respondent healthcare facility/ **Kituo cha Afya cha mtoa huduma:** _________

Respondent primary work location/**Kitengo anachofanyia kazi mtoa huduma:**

□ EMD/OPD, **Idara ya dharura /Wagonjwa wa nje.**

□ Inpatient ward/ **Wagonjwa wa ndani**

□ Follow-up cardiac clinic/ **Marudio kliniki ya moyo.**

□ Other, specify/**Nyingine, Taja/ainisha :** ___________

**Section 1: Affective Attitude/ *Sehemu ya 1: Mtazamo***

1. I am interested in participating in a quality improvement project to improve MI care at my facility/**Nipo tayari kushiriki katika uboresha wa mikakati ya huduma ya mshtuko wa moyo katika kituo changu:**

□ Strongly Agree/**Nakubali kabisa** □ Agree/**Nakubali**  □ Neutral/don’t know/**Sijui** □ Disagree/**Sikubali**  □ Strongly Disagree/ **Sikubali kabisa.**

1a. MI is a common condition among patients at my facility/**Mshituko wa moyo ni tatizo linalowapata miongoni mwangojwa katika kituo changu:**

□ Strongly Agree/**Nakubali kabisa** □ Agree/**Nakubali** □ Neutral/Don’t know/**Sijui**  □ Disagree/**Sikubali**  □ Strongly Disagree/**Sikubali kabisa**

2. Improvements are needed to current MI care pathways at my facility/**Uboreshaji unahitajika kwa sasa katika nyanja ya utoaji huduma ya mshituko wa moyo katika kituo changu:**

□ Strongly Agree /**Nakubali kabisa** □ Agree /**Nakubali**  □ Neutral/Don’t know/**Sijui**  □ Disagree/**Sikubali**  □ Strongly Disagree/**Sikubali kabisa**

3. I believe that there are things providers can do to improve MI care and outcomes for my patients/**Naamini kwamba kuna vitu vinaweza kufanyika na watoa huduma katika kuboresha huduma ya mshtuko wa moyo nakuleta matokeo kwa wagonjwa wangu.**

□ Strongly Agree/**Nakubali kabisa** □ Agree/**Nakubali**  □ Neutral/don’t know/**Sijui**  □ Disagree/**Sikubali**  □ Strongly Disagree/**Sikubali kabisa**

4. Most providers at my facility have received adequate training to provide high-quality MI care to our patients/**Idadi kubwa ya watoa huduma katika kituo changu wamepata mafunzo ya kutosha katika kutoa huduma bora ya kiwango cha juu katika kuwahudumia wagonjwa wetu:**

□ Strongly Agree/**Nakubali kabisa** □ Agree/**Nakubali**  □ Neutral/don’t know/**Sijui**  □ Disagree/**Sikubali**  □ Strongly Disagree/**Sikubali kabisa**

5. I feel most providers at my facility are competent in diagnosing MI by using patient history, ECG, and laboratory data/**Nahisi watoa huduma wengi katika kituo changu wanaujuzi katika kugundua shida ya mstuko wa moyo, kwakuchukua historia kwa mgonjwa, kutumia kipimo cha umeme wa moyo ECG na taarifa kutoka maabara:**

□ Strongly Agree/**Nakubali kabisa** □ Agree /**Nakubali**  □ Neutral/don’t know/**Sijui**  □ Disagree /**Sikubali**  □ Strongly Disagree/**Sikubali kabisa**

6. I feel most providers at my facility are competent in treating MI in the ED/hospital setting/**Ninahisi watoa huduma wengi wanauwezo wa kutibu tatizo la mshtuko wa moyo katika idara ya dharura/hosipital kwa ujumla:**

□ Strongly Agree/**Nakubali kabisa** □ Agree/**Nakubali**  □ Neutral/Don’t know/**Sijui**  □ Disagree/**Sikubali**  □ Strongly Disagree/**Sikubali kabisa**

6a. I think the protocol for treating MI patients presenting at my facility is easily accessible/**Nafikiri upatikanaji wa miongozo ya kutibu wagonjwa wa mshtuko wa moyo wanaofika hospitalini ni rahisi sana kupatikana:**

□ Strongly Agree/**Nakubali kabisa** □ Agree/**Nakubali**  □ Neutral/Don’t know/**Sijui** □ Disagree/**Sikubali**  □ Strongly Disagree/**Sikubali kabisa**

7. I feel most providers at my facility are competent in prescribing the best secondary preventative medications for MI when discharging patients with MI/**Nafikiri idadi kubwa ya watoa huduma katika kituo changu wanauwezo wa kutosha katika kuwaandikia dawa za kuzuia athari za mshtuko wa moyo pale waporuhusiwa:**

□ Strongly Agree/**Nakubali kabisa** □ Agree/**Nakubali**  □ Neutral/Don’t know/**Sijui** □ Disagree/**Sikubali**  □ Strongly Disagree/**Sikubali kabisa**

8. I feel most providers at my facility are competent in providing guidance to patients with MI about lifestyle and dietary changes to prevent further complications of their disease/**Nahisi watoa huduma katika kituo changu, wanaujuzi wa kutosha katika kuwapatia miongozo kwa wangojwa wa mshtuko wa moyo, juu ya mabadiliko ya kimaisha, vyakula ilikupunguza athari kubwa zitakazoweza kusababishwa na magonjwa yao:**

□ Strongly Agree/ **Nakubali kabisa** □ Agree/**Nakubali**  □ Neutral/Don’t know/**Sijui**  □ Disagree/**Sikubali** □ Strongly Disagree/**Sikubali Kabisa.**

9. I believe most patients with MI at my facility are discharged with prescriptions for the correct secondary preventative medications/ **Ninaamini wagonjwa wengi ambao wanashida ya mshtuko wa moyo katika kituo changu huwa wa naruhusiwa na dawa sahihi ambazo zinazuia athari zinazotokana mshituko wa moyo:**

□ Strongly Agree/**Nakubali kabisa** □ Agree/**Nakubali**  □ Neutral/Don’t know/**Sijui**  □ Disagree/**Sikubali** □ Strongly Disagree/**Sikubali kabisa**

10. I believe most patients with MI at my facility receive proper dietary and lifestyle counseling prior to discharge/**Ninaamini wagojwa wengi katika kituo changu wanapata ushauri sahihi katika vya kula na mabadiliko ya kimaisha kabla ya kuruhusiwa:**

□ Strongly Agree/**Nakubali kabisa** □ Agree/ **Nakubali**  □ Neutral/Don’t know/**Sijui**  □ Disagree/**Sikubali**  □ Strongly Disagree/**Sikubali kabisa**

11. I believe most patients with MI at my facility receive proper outpatient follow-up/**Naamini wagonjwa wengi wenye mshtuko wa moyo wanapata ufuatiliaji sahihi wakati wa marudio:**

□ Strongly Agree/**Nakubali kabisa** □ Agree/**Nakubali**  □ Neutral/Don’t know/**Sijui**  □ Disagree/**Sikubali**  □ Strongly Disagree/**Sikubali kabisa**

13. I think patients with MI need more education about the disease/**Nafikiri wangojwa wa mshituko wa moyo wanahitaji elimu Zaidi juu ya ugonjwa huo:**

□ Strongly Agree/**Nakubali kabisa** □ Agree/**Nakubali** □ Neutral/Don’t know/**Sijui**  □ Disagree/**Sikubali**  □ Strongly Disagree/**Sikubali kabisa**

14. I believe patients with MI will take their prescriptions at home and follow lifestyle guidance they receive:/**Naamini wagonjwa wenye mshtuko wa moyo watatumia dawa zao wakiwa nyumbani na kufuatilia miongozo ya mabadiliko ya kimaisha kama waliovyopewa:**

□ Strongly Agree /**Nakubali kabisa** □ Agree/ **Nakubali** □ Neutral/Don’t know/**Sijui** □ Disagree /**Sikubali**  □ Strongly Disagree/**Sikubali kabisa**

15. I believe patients with MI will attend follow-up appointments that are arranged for them: **Naamini wagonjwa wenye mshtuko wa moyo watahudhuria tarehe zao za marudio kama zilivyopangwa:**

□ Strongly Agree/**Nakubali kabisa** □ Agree /**Nakubali**  □ Neutral/Don’t know/**sijui**  □ Disagree/**Sikubali**  □ Strongly Disagree/**Sikubali kabisa**

16. I think additional training for providers about MI diagnosis and ECG interpretation would improve MI care at my facility:**Ninafikiri mafunzo ya ziada kwa watoa huduma juu ya utambuzi wa ugonjwa wa mshtuko wa moyo na kutafsiri kipimo cha umeme wa moyo utaboresha huduma ya moyo katika kituo changu**

□ Strongly Agree/ **Nakubali kabisa** □ Agree/**Nakubali** □ Neutral/Don’t know /**sijui**  □ Disagree / **Sikubali**  □ Strongly Disagree/**Sikubali kabisa**

17. I think additional training for providers about MI treatment guidelines would improve MI care at my facility: **Nafikiri mafunzo ya ziada kwa watoa huduma kuhusu miongozo ya matibabu ya mshtuko wa moyo yataboresha matibabu ya moyo katika kituo changu**

□ Strongly Agree/**Nakubali kabisa** □ Agree /**Nakubali** □ Neutral/Don’t know /**Sijui**  □ Disagree /**Sikubali** □ Strongly Disagree/**Sikubali kabisa**

18. I think patient education would improve MI treatment guidelines at my facility: **Nafikiri elimu kwa wagonjwa itaboresha miongozo ya matibabu ya moyo katika kituo changu:**

□ Strongly Agree/**Nakubali kabisa** □ Agree/**Nakubali**  □ Neutral/Don’t know /**Sijui**  □ Disagree/**Sikubali**  □ Strongly Disagree/**Sikubali kabisa**

19. I think having a checklist to remind providers of MI acute treatment guidelines would improve MI care for patients in the ED or inpatient wards at my facility: **Nafikiri kuwa na muongozo ya kuwakumbusha watoa huduma juu ya matibabu ya dhadhura ya mshtuko itaboresha huduma kwa wagonjwa wa mshukto wa moyo katika idara ya dhadhura au waliolazwa katika kituo changu:**

□ Strongly Agree/**Nakubali kabisa** □ Agree / **Nakubali** □ Neutral/Don’t know /**Sijui** □ Disagree/ **Sikubali**  □ Strongly Disagree/**Sikubali kabisa**

20. I think having a discharge checklist to remind providers of secondary preventative guidelines would improve discharge care for MI patients at my facility: **Nafikiri kuwa na orodha hakiki kuwakumbusha watoa huduma juu ya miongozo itakayopunguza athari, itaboresha huduma wakati mgonjwa anaporuhusiwa katika kituo changu:**

□ Strongly Agree/**Nakubali kabisa** □ Agree/**Nakubali** □ Neutral/Don’t know /**Sijui**  □ Disagree/**Sikubali**  □ Strongly Disagree/**Sikubali kabisa**

21. I think reminders built into the electronic medical record would help improve MI care at my facility:**Nafikiri kuwepo kwa vifaa kumbusho vya kielectroniki kwenye mfumo wa utunzaji kumbukumbu za kiafya utasaidia kuboresha huduma za mshtuko wa moyo katika kituo change:**

□ Strongly Agree /**Nakubali kabisa** □ Agree/**Nakubali**  □ Neutral/Don’t know/**Sijui**  □ Disagree /**Sikubali** □ Strongly Disagree/**Sikubaki kabisa**

21a. I think order sets built into the EMR/paper record would help improve MI care at my facility:/ **Nafikiri uwepo wa karatasi za kumbukumbu katika idara za dhadhura kutasaidia kuboresha huduma ya mshtuko wa moyo katika kituo changu:**

□ Strongly Agree/**Nakubali kabisa** □ Agree /**Nakubali**  □ Neutral/Don’t know /**Sijui** □ Disagree/**Sikubali**  □ Strongly Disagree/**Sikubali kabisa**

22. I think the process of follow-up in a designated cardiac clinic for MI patients works well at my facility:/ **Nafikiri taratibu zilizowekwa za ufuatiliaji wa marudio katika kliniki ya moyo unafanya kazi vizuri katika kituo changu:**

□ Strongly Agree /**Nakubali kabisa** □ Agree/**Nakubali**  □ Neutral/Don’t know /**Sijui** □ Disagree /**Sikubali**  □ Strongly Disagree/**Sikubali kabisa**

23. I think having an audit mechanism whereby providers receive feedback about their individual MI care in a supportive/nonjudgmental manner would improve MI care at my facility: / **Nafikiri kuwepo na mfumo rafiki wa ukaguzi ambapo watoa huduma wanapokea mrejesho kuhusiana na huduma wanazotoa juu ya mshtuko wa moyo itaboresha huduma za mshtuko wa moyo katika kituo change:**

□ Strongly Agree/**Nakubali kabisa** □ Agree /**Nakubali**  □ Neutral/Don’t know /**Sijui** □ Disagree /**Sikubali**  □ Strongly Disagree/**Sikubali kabisa**

23a. If you think it would be helpful to have an audit/feedback mechanism, would you prefer to receive this feedback at the individual level or receive general feedback as a department**? Kama unafikiri kuwepo kwa mfumo rejesho kutasaidia, je utapendelea kupata mrejesho binafsi au kupata mrejesho kwa ujumla kama idara?**

□ Individual /**Binafsi**  □ Department / **Idara**  □ Both /**Vyote**  □ Unsure/ **Sina uhakika**

24. I think nurse-driven protocols where certain aspects of MI care happen (such as ordering ECG, giving aspirin) occur without physician orders would improve MI care at my facility: **Nafikiri kuwepo na miongozo itakayo muongoza muuguzi katika vipengele mbalimbali vya mshtuko wa moyo ( kama vile kuagiza ufanyaji wa kipimo cha umeme wa moyo, kutoa dawa ya asprine) kutokea bila agizo la daktari itaboresha huduma ya mshtuko wa moyo katika kituo change:**

□ Strongly Agree /**Nakubali kabisa** □ Agree /**Nakubali**  □ Neutral/Don’t know /**sijui** □ Disagree /**Sikubali**  □ Strongly Disagree/**Sikubali kabisa**

24a. I think ensuring certain basic MI medications (aspirin, heparin) are available in-house would improve MI care at my facility/ **Nafikiri kuhakikisha upitikanagi wa dawa mihumu za mshtuko wa moyo ( aspirine na /heparin) zitasaidia kuboresha huduma za mshtuko wa moyo katika kituo change:**

□ Strongly Agree/**Nakubali kabisa** □ Agree/**Nakubali**  □ Neutral/Don’t know /**Sijui**  □ Disagree **Sikubali**  □ Strongly Disagree/**Sikubali kabisa**

25. Of all potential interventions to improve MI care at my facility, I think the most impactful would be/**Katika mikakati yote madhubuti ya kuboresha matibabu ya mshtuko wa moyo katika kituo changu, nafikiri itakayoleta mabadiliko makubwa/chanya ni**:

□ Patient Education /**Elimu kwa wagonjwa** □ Additional Provider training /**Mafunzo ya ziada kwa** **watoa huduma** □ Checklists / **orodha hakiki** □ Standardized follow-up / **ufuatiliaji bora wa marudio**
□ Reminders in the electronic medical record / **Kifaa kumbushi cha kieletroniki katika mfumo wa utunzaji wa kumbukumbu** □ Audit/feedback /**Ukaguzi/Mrejesho** □ Nurse-drive protocols/**Miongozo ya kumuongoza muuguzi**
□ Order Sets / **Maagizo**  □ Stocking medications in-house / **Upatikanaji wa dawa wakati wote** □ Other : **Nyingine**_______ (Specify)(**Taja/Anisha)**

26. I have the following additional suggestions for improving MI care at my facility/**Yafuatazo ni mapendekezo ya ziada yatakoyoboresha huduma ya mshtuko wa moyo katika kituo changu** ____________

**Section 2: Burden/ *sehemu ya 2: vikwazo***

27. Relative to other issues at my facility, efforts to improve MI care at my facility should be a priority/**Tofauti na changamoto nyingine katika kituo changu, Jitihada zinaitajika kupewa kipaumbele ili kuboresha huduma za mshutko wa moyo:**

□ Strongly Agree /**Nakubali kabisa** □ Agree /**Nakubali**  □ Neutral/Don’t know / **Sijui** □ Disagree /**Sikubali** □ Strongly Disagree/**Sikubali kabisa**

28. Additional MI training for providers would be a burden on my time/ **Mafunzo ya ziada juu ya mshtuko wa moyo kwa watoa huduma yatakuwa kikwazo katika mda wangu:**

□ Strongly Agree /**Nakubali kabisa** □ Agree /**Nakubali**  □ Neutral/Don’t know /**Sijui**  □ Disagree /**Sikubali**  □ Strongly Disagree/**Sikubali kabisa**

29. Checklists, reminders, and audit/feedback about MI care would be disruptive and burdensome to me, and would interfere with my many other patient care responsibilities/ **Orodha hakiki, ukumbusho, na ukaguzi/mrejesho kuhusiana na mshtuko wa moyo vitakuwa kero na kikwazo kwangu na itaingilia na majukumu mengine ya utoaji huduma kwa wagonjwa:**

□ Strongly Agree /**Nakubali kabisa** □ Agree/**Nakubali** □ Neutral/Don’t know /**Sijui**  □ Disagree / **Sikubali**  □ Strongly Disagree/**Sikubali kabisa**

30. I do not have time to participate in an MI quality improvement project/**Sina muda wa kushiriki katika mikakati ya kuboresha huduma ya kuboresha juu ya mshtuko wa moyo:**

□ Strongly Agree/ **Nakubali kabisa** □ Agree / **Nakubali**  □ Neutral/Don’t know /**Sijui**  □ Disagree / **Sikubali**  □ Strongly Disagree/**Sikubali kabisa**

**Section 3: Ethicality/*Sehemu ya 3: Maadili***

31. Current MI care at my facility is already adequate, and quality improvement efforts are not needed/ **Kwa sasa huduma ya mshtuko wa moyo inajitosholeza, na juhudi za kuboresha huduma ya mshtuko wa moyo haziitajiki:**

□ Strongly Agree /**Nakubali kabisa** □ Agree /**Nakubali**  □ Neutral/Don’t know/**Sijui**  □ Disagree /**Sikubali** □ Strongly Disagree/**sikubali kabisa**

32. Providing high-quality MI care at my facility is important to me/ **Kwangu utoaji huduma bora kwa mshtuko wa moyo ni muhimu katika kituo change:**

□ Strongly Agree/**Nakubali kabisa** □ Agree/**Nakubali**  □ Neutral/Don’t know /**Sijui**  □ Disagree /**Sikubali**  □ Strongly Disagree/**Sikubali kabisa**

33. Efforts to improve MI care at my facility will have other unintended negative consequences/**Juhudi za kuboresha huduma ya mshtuko wa moyo italeta matokeo yasiotarajiwa katika kituo change:**

□ Strongly Agree/**Nakubali kabisa** □ Agree /**Nakubali**  □ Neutral/Don’t know /**Sijui**  □ Disagree /**Sikubali** □ Strongly Disagree/**Sikubali kabisa**

34. If provider chose “Agree” or “Strongly agree”, please elaborate about these unintended negative consequences/ **Kama mtoa huduma atachagua “nakubali “au” Nakubali kabisa”, Tafadhali elezea hayo matokeo yasiotarajiwa**___________________

35. Do you have any other thoughts about improving MI care that you would like to share with us? **Je, unamapendekezo yoyote juu ya kuboresha huduma ya mshtuko wa moyo utakayopenda kutushirikisha?** __________________________

36. Does your facility have a current MI care protocol? **Je, kituo chako kina muongozo wa sasa unaotumika katika hutoaji huduma ya mshtuko wa moyo?**

□ Yes /**Ndiyo**  □ No /**Hapana** □ Don’t know /**Sijui**

37. Why do you think some providers do not follow the MI care protocol (choose all that apply)? **Kwanini unafikiri baadhi wa watoa huduma hawafuati miongozo katika utoaji wa mshtuko wa moyo(chagua vyote vinavyohusika)?**

□ They don’t know it exists / **Hawatambui kama ipo.** □ They forget /**Wanasahau**  □ The protocol is difficult to find /**ni ngumu kupatikana kwa miongozo**

□ The protocol is too hard/complicated / **Miongozo ni migumu kutumia/inachanganya** □ They don’t have enough training about MI/ **Hawana mafunzo ya kutosha juu ya mshutko ya moyo**

□ Other/**Nyingine** specify:**Taja/ainisha** ___________
