# Supplementary material for: Provider attitudes towards quality improvement for myocardial infarction care in northern Tanzania
Source: PLOS Glob Public Health. 2024 Apr 4;4(4):e0003051. doi: 10.1371/journal.pgph.0003051 (PMC10994299; doi:10.1371/journal.pgph.0003051)
Supplement: S1 Table — (DOCX) [file pgph.0003051.s004.docx]

**Table A: Provider affective attitudes towards MI care, northern Tanzania, 2022**

| **Statement** | **Nurses: Strongly Agree/agree n (%)**  **N=82** | **Physicians/Administrators**  **Strongly Agree/agree n (%)**  **N=58** | **P-value** |
| --- | --- | --- | --- |
| **I am interested in participating in a quality improvement project to improve MI care at my facility** | 82(100) | 57(98.3) | N/A |
| **MI is a common condition among patients at my facility** | 81 (98.8) | 54(93.1) | 0.075 |
| **Improvements are needed to current MI care pathways at my facility** | 82(100) | 58(100) | N/A |
| **I believe that there are things providers can do to improve MI care and outcomes for my patients** | 82(100) | 58(100) | N/A |
| **Most providers at my facility have received adequate training to provide high-quality MI care to our patients** | 42(51.2) | 22(37.9) | 0.120 |
| **I feel most providers at my facility are competent in diagnosing MI by using patient history, ECG, and laboratory data** | 53(64.6) | 31(53.5) | 0.183 |
| **I feel most providers at my facility are competent in treating MI in the ED/hospital setting** | 55(67.0) | 33(56.9) | 0.220 |
| **I think the protocol for treating MI patients presenting at my facility is easily accessible** | 58(70.7) | 38(65.5) | 0.513 |
| **I feel most providers at my facility are competent in prescribing the best secondary preventative medications for MI when discharging patients with MI** | 61(74.4) | 37(64.5) | 0.178 |
| **I feel most providers at my facility are competent in providing guidance to patients with MI about lifestyle and dietary changes to prevent further complications of their disease** | 62(75.6) | 40(63.8) | 0.384 |
| **I believe most patients with MI at my facility are discharged with prescriptions for the correct secondary preventative medications** | 71(86.6) | 46(79.3) | 0.252 |
| **I believe most patients with MI at my facility receive proper dietary and lifestyle counseling prior to discharge** | 61(74.4) | 36(62.0) | 0.120 |
| **I believe most patients with MI at my facility receive proper outpatient follow-up** | 51(62.2) | 27(46.5) | 0.004 |
| **I think patients with MI need more education about the disease** | 82 (100) | 58 (100) | N/A |
| **I believe patients with MI will take their prescriptions at home and follow lifestyle guidance they receive** | 52 (63.4) | 29(50) | 0.616 |
| **I believe patients with MI will attend follow-up appointments that are arranged for them** | 58 (70.7) | 38 (65.5) | 0.650 |
| **I think the process of follow-up in a designated cardiac clinic for MI patients works well at my facility** | 54 (65.8) | 44 (75.9) | 0.600 |

**Table B: Provider perceptions of the burden associated with a quality improvement program for MI care, northern Tanzania, 2022**

| **Statement** | **Nurses: Strongly Agree/agree n (%) N=82** | **Physicians/Administrators**  **Strongly Agree/agree n (%)**  **N=58** | **P-values** |
| --- | --- | --- | --- |
| **Relative to other issues at my facility, efforts to improve MI care at my facility should be a priority** | 78 (95.1) | 54 (93.1) | 0.816 |
| **Additional MI training for providers would be a burden on my time** | 10 (12.2) | 9 (15.5) | 0.069 |
| **Checklists, reminders, and audit/feedback about MI care would be disruptive and burdensome to me, and would interfere with my many other patient care responsibilities** | 11 (13.4) | 11 (19) | 0.374 |
| **I do not have time to participate in an MI quality improvement project** | 7 (8.5) | 6 (10.3) | 0.033 |

**Table C: Perceived effectiveness of various MI quality improvement strategies among providers, northern Tanzania, 2022**

| **Statement** | **Nurses: Strongly Agree/agree n (%) N=82** | **Physicians/Administrators**  **Strongly Agree/agree n (%)**  **N=58** | **P-value** |
| --- | --- | --- | --- |
| **I think additional training for providers about MI diagnosis and ECG interpretation would improve MI care at my facility** | 80 (97.5) | 58 (100) | N/A |
| **I think additional training for providers about MI treatment guidelines would improve MI care at my facility** | 81 (98.8) | 58 (100) | N/A |
| **I think patient education would improve MI treatment guidelines at my facility** | 81 (98.8) | 58 (100) | N/A |
| **I think having a checklist to remind providers of MI acute treatment guidelines would improve MI care for patients in the ED or inpatient wards at my facility** | 80 (97.6) | 58 (100) | N/A |
| **I think having a discharge checklist to remind providers of secondary preventative guidelines would improve discharge care for MI patients at my facility** | 79 (96.3) | 58 (100) | N/A |
| **I think reminders built into the electronic medical record would help improve MI care at my facility** | 79 (96.3) | 55 (94.8) | 0.681 |
| **I think order sets built into the EMR/paper record would help improve MI care at my facility** | 71 (86.5) | 51 (87.9) | 0.815 |
| **I think having an audit mechanism whereby providers receive feedback about their individual MI care in a supportive/nonjudgmental manner would improve MI care at my facility** | 76 (92.7) | 56 (96.5) | 0.816 |
| **I think nurse-driven protocols where certain aspects of MI care happen (such as ordering ECG, giving aspirin) occur without physician orders would improve MI care at my facility** | 53(64.6) | 39 (67.2) | 0.687 |
| **I think ensuring certain basic MI medications (aspirin, heparin) are available in-house would improve MI care at my facility** | 76 (92.7) | 51 (88) | 0.158 |

**Table D: Provider perceptions of the ethicality of an MI quality improvement intervention in northern Tanzania, 2022**

| **Question** | **Nurses: Strongly Agree/agree, n (%)**  **N=82** | **Physicians/Administrators**  **Strongly Agree/agree, n (%)**  **N=58** | **P-value** |
| --- | --- | --- | --- |
| **Current MI care at my facility is already adequate, and quality improvement efforts are not needed** | 5 (6.1) | 5 (8.6) | 0.358 |
| **Providing high-quality MI care at my facility is important to me** | 82 (100) | 56 (98.3) | N/A |
| **Efforts to improve MI care at my facility will have other unintended negative consequences** | 8 (9.8) | 6 (10.3) | 0.663 |
